# Perinatal Outcomes in Foetuses with Increased Nuchal Translucency and Normal Karyotype: A Retrospective Cohort Study from the United Arab Emirates

**DOI:** 10.3390/jcm12196358

**Published:** 2023-10-04

**Authors:** Howaida Khair, Serene Hilary, Shamsa Al Awar, Kornelia Zareba, Sara Maki, Gehan Sayed, Sharon Mutare, Ayman W. El-Hattab, Ali Hussein Al Ibrahim

**Affiliations:** 1 Department of Obstetrics and Gynecology, College of Medicine and Health Sciences, United Arab Emirates University, Al Ain P.O. Box 15551, United Arab Emirateskzareba@uaeu.ac.ae (K.Z.);; 2 Department of Nutrition and Health, College of Medicine and Health Sciences, United Arab Emirates University, Al Ain P.O. Box 15551, United Arab Emirates; serene_h@uaeu.ac.ae (S.H.);; 3 Fetal Maternal Unit, Kanad Hospital, Al Ain P.O. Box 1016, United Arab Emirates; 4 Department of Clinical Sciences, College of Medicine, University of Sharjah, Sharjah P.O. Box 27272, United Arab Emirates

**Keywords:** nuchal translucency, enlarged NT, perinatal outcomes, amniocentesis, chorionic villus sampling, karyotype, congenital anomalies, microarray

## Abstract

This retrospective case-controlled study analysed the outcome of pregnancies with first-trimester enlarged nuchal translucency (NT) and a normal karyotype. A total of 479 pregnancies with first-trimester NT measurements were grouped as control (370 cases; normal NT) and study (109 cases; enlarged NT, ≥95th percentile; with normal karyotype). Adverse outcomes included miscarriage, intrauterine foetal death, termination of pregnancy, neonatal death, and structural/chromosomal/genetic abnormalities. The study was conducted between June 2016 and June 2022 at the Foetal Maternal Unit of Kanad Hospital, UAE. Overall, the live birth rate in the study group was significantly lower (74.3%) compared to the control (94.1%, *p* < 0.001). All pregnancy outcomes of this group significantly differed compared to the control. The observed miscarriage level was 9.2% (vs. 1.1%, *p* < 0.001), intrauterine foetal death was 2.8% (vs. 0%, *p* = 0.001), spontaneous preterm birthwas 11% (vs. 4.9%, *p* = 0.020), and termination of pregnancy was 3.7% (vs. 0%, *p* < 0.001). The presence of foetal abnormalities was also significantly higher in the enlarged NT group at 21% (vs. 3.3%, *p* < 0.001). Results indicate that enlarged NT is associated with adverse pregnancy outcomes even when the karyotype is normal. Based on these results, a comprehensive review of the guidelines for counselling and managing pregnancies with enlarged NT and a normal karyotype is recommended.

## 1. Introduction

Nuchal translucency (NT) is a subcutaneous fluid accumulation in the posterior aspect of the foetal neck, which is used as a marker for aneuploidy in the first trimester [1]. Ultrasonography can detect NT from the first week for up to 13 weeks of gestation. NT measurements are a vital imaging biomarker for aneuploidy in the first trimester [1]. The use of NT measurements as a biomarker to predict foetal aneuploidy was reported as early as 1992 [1,2]. Initially, NT measurement was used as a solo biomarker of foetal chromosomal abnormalities. The screening of foetal autosomal trisomies 21, 18, and 13 and significant structural anomalies were further refined with combined first-trimester screening (FTS) [3]. The combined FTS comprises maternal age, detailed ultrasonography measuring the crown–rump length (CRL) and the NT of the foetus at 11 to 13 weeks of gestation, and maternal serum biomarkers-assessment. The maternal serum biomarkers commonly used are free β-human chorionic gonadotropin and pregnancy-associated plasma protein [3]. Enlargement NT measurements are linked to conditions such as trisomy 21, Turner syndrome, various chromosomal anomalies, numerous foetal malformations and genetic syndromes [4,5,6,7]. The occurrence of these abnormalities is primarily connected to the thickness of NT rather than its visual characteristics [8]. At its core, NT is part of normal foetal development, and various factors influence its thickness. This measurement is abnormal when it exceeds an accepted cut-off level [9]. Generally, an enlarged NT is characterised by a measurement exceeding the 95th percentile [10]. This definition is applied even if the fluid accumulation has septated, and whether NT is localised solely in the neck region or encompasses the entire foetus [11]. NT thickness tends to rise in correlation with foetal CRL. According to a large-cohort study involving 96,127 pregnancies [12], the median NT thickness at a CRL of 45 mm was 1.2 mm, while the 95th percentile reached 2.1 mm. Similarly, at a CRL of 84 mm, the median NT thickness was 1.9 mm, and the 95th percentile reached 2.7 mm. This study noted that the 99th percentile remained relatively stable with changes in CRL, hovering around 3.5 mm [12].

Debates continue about whether the 95th or 99th percentile should be used as a cut-off for increased NT measurements. The consensus is that an NT measurement above the 95th percentile (3.5 mm) is an enlarged NT [9]. However, the definition of these cut-offs can vary across populations. Currently, NT measurements are used in most populations as a means for first-trimester screening for chromosomal abnormalities. When women are counselled on first-trimester screening, they must be informed about the risk of chromosomal or structural anomalies and even genetic syndromes. In foetuses with enlarged nuchal translucency (NT), the likelihood of experiencing an adverse outcome, including chromosomal abnormalities and other adverse outcomes such as foetal mortality and postnatal death, increases in tandem with NT thickness, as reported in numerous studies [13,14,15]. This risk varies from about 5% for NT measurements falling between the 95th percentile and 3.4 mm [16]. The risk increases to 30% for NT measurements ranging from 3.5 to 4.4 mm, 50% for NT values in the 4.5 to 5.4 mm range and surges to 80% for NT measurements equal to or exceeding 5.5 mm [16].

Moreover, based on the literature, the overall chance of adverse outcomes also varies substantially according to the cohort characteristics. In cohort studies of foetuses with enlarged NT, the adverse outcomes range 3–6% [17]. However, the percentage was significantly higher in other cohorts with foetuses of high-risk pregnancies, at about 20% [17]. Due to the variability in these outcomes across the different populations, data from population-specific cohort studies can aid in constructing better diagnostic and counselling practices. In general practice, parents are counselled that the probability of giving birth to a baby without significant abnormalities stands at approximately 97% for NT measurements below the 95th percentile and 93% for NT readings falling between the 95th and 99th percentiles [11]. However, in the absence of chromosomal anomalies with enlarged NT, there is a knowledge gap when it comes to highlighting the risk of adverse pregnancy outcomes in patients in the United Arab Emirates, and there are no existing local guidelines on patient counselling for cases with enlarged NT and normal karyotype. The current study aims to address this knowledge gap.

## 2. Materials and Methods

### 2.1. Study Design and Outcomes

This was a retrospective cohort study conducted at the Foetal Maternal Unit at Kanad Hospital in Al Ain, United Arab Emirates. Research Ethics approval for the study was obtained from the Kanad Hospital Research Ethics Committee (approval code 2012.15). Data from all pregnant women who consulted the Foetal Maternal Unit from June 2016 to June 2022 were reviewed and added to the study based on the inclusion and exclusion criteria. The study population were selected from pregnancies that received first-trimester screening for foetal aneuploidy at 11–14 weeks of gestation at the clinic. In the Foetal Maternal Unit of Kanad Hospital, the combined FTS and NT measurements are performed by physicians’ sonographers accredited by the Foetal Medicine Foundation-UK (FMF-UK).

All NT measurements ≥2.5 mm recorded in the study period were retrieved from the Astraia software database. Enlarged NT was defined as a measurement ≥95th percentile by CRL-adjusted percentiles (CRL range 45–84 mm). Only cases with known pre- and postnatal information, and results of detailed ultrasound examination, karyotyping by amniocentesis or chorionic villous sampling (CVS), delivery reports, and neonatal physical examination of babies with visible abnormalities at birth were considered. In Kanad Hospital, CVS or amniocentesis is offered to women if the combined test risk is 1:250 or more. For prenatal screening, karyotyping and chromosomal microarray analysis are performed to detect chromosomal abnormalities. The inclusion criteria for the study group were women with NT measurements ≥95% with a normal result for chorionic villus sampling or amniocentesis; singleton pregnancy; pregnant women without inheritable risk, tumour or pre-eclampsia; and women with no history of chronic medical diseases such as diabetes mellitus, renal or autoimmune diseases.

The participants for the control group were selected from a consecutive series of pregnancies with normal NT where perinatal data were available and delivered within the study period. The general exclusion criteria in both control and study groups were pregnancies with twins, a history of chronic conditions such as diabetes mellitus, hypertension, autoimmune and renal diseases, and access to delivery details. Adverse pregnancy outcomes were miscarriage, intrauterine foetal death, termination of pregnancy (TOP), neonatal death, structural defects, and genetic disorders. Miscarriage was defined as foetal loss at <22 weeks and intrauterine foetal death at ≥22 weeks. Spontaneous preterm birth was defined as spontaneous birth under 37 weeks of gestation. Favourable pregnancy outcomes were healthy live-born babies without structural, genetic, or chromosomal abnormalities diagnosed at postnatal follow-up.

### 2.2. Statistical Analyses

Statistical analyses for the data were performed using GraphPad Prism software version 9.1.0. Model assumptions for the continuous variables were checked by the D’Agostino and Pearson and Shapiro–Wilk tests. When model assumptions were violated, the non-parametric Mann–Whitney U test determined the difference between the control and study groups. A t-test was used to compare the data study and control group data in all other cases. The chi-square or Fisher’s exact test was used for categorical variables. Continuous variables are presented as mean ± SD, and categorical variables are presented as percentages and counts. Binary logistic regression was used to determine the factors influencing adverse pregnancy outcomes in the population. Potential covariates associated with no-result embryos adjusted for in the model included age, BMI and NT as continuous variables and consanguinity, second-trimester scan result and parity as categorical variables. All variables were entered using a forced entry method. All the predictor variables were tested in one block to assess their predictive ability while controlling for other predictors in the model. Spearman’s correlation coefficients calculated the association between various factors and adverse pregnancy outcomes and detected anomalies in neonates. A *p*-value of ≤ 0.05 was considered statistically significant.

## 3. Results

### 3.1. Study Population

**Figure 1:**
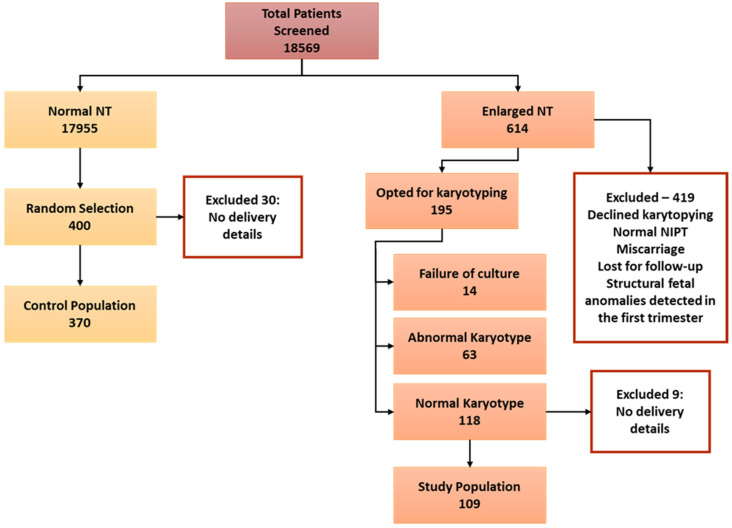
Illustration of study population screening and selection.

In the study period, 18,569 patient files were screened in the database, where 17,955 cases recorded normal NT measurements and 614 recorded enlarged NT defined as ≥95% (Figure 1). Among the cases with enlarged NT measurements, 419 were excluded since they did not opt for CVS or amniocentesis, had a miscarriage, had abnormal non-invasive prenatal tests, lost for follow-up or had foetal structural anomalies in the first-trimester scan. Among the remaining 195 patients, 63 had an abnormal karyotype, and 14 were excluded due to failure of culture for karyotypes. The population with enlarged NT and normal karyotype of 118 participants were further screened to include only patients with delivery details. The final number in the enlarged NT with normal karyotype was 109. For the control population, 400 patients were randomly selected from the identified pool of 17,955 patients with normal NT. A total of 30 cases were excluded as there were no delivery details, and the final number included in the control group was 370.

Table 1 shows demographic information and patient characteristics. The mean maternal age of the control and study groups were 32.28 ± 5.1 and 33.78 ± 6.1 years, respectively. There was a significant difference in age between the groups (*p* = 0.0077). The BMI of the study participants were comparable between the two groups (*p* = 0.2466) and ranged from 28.06 ± 5.73 and 27.14 ± 4.60 in the control and study groups. Similarly, the parity between the two groups was consistent, with 66.2% and 67.0% reporting <4 and 33.8% and 33% reporting ≥4 in the control and study groups, respectively. Most pregnancies in the study were spontaneous (97.3% control, 99.1% study). There were only 11 cases of pregnancy due to assisted reproductive technique in the whole population, 10 in control and 1 in the study group. Another noteworthy observation was that the consanguinity in the population was also consistent, with 36.3% and 33.9% in control and study groups. The reported consanguinity included first-degree cousins (28.5% control, 29.4% study group) and second-degree cousins (7.1% control and 4.6% study group). The mean CRL in the whole population was 64.69 mm and did not vary significantly between the two groups (*p* = 0.951). The NT measurements in the population ranged 1–15 mm with a mean value of 2.34 mm. The recorded NT in the control group was 1.747 ± 0.3 mm, and in the study group was 4.368 ± 1.9 mm (*p* = <0.001).

### 3.2. Pregnancy and Delivery Outcomes

All pregnancy outcomes in the study significantly varied between the control and study group (Table 2). In the study population of 479 participants, second-trimester scan data were missing for 13 cases, because the scans were performed in another medical centre or due to miscarriage. A total of 7.71% of the population had abnormal anatomy scan results in the second trimester, and there was a significant difference in the observations between the control and study groups. Abnormal results in the second-trimester anatomy scan were significantly higher at 28.2% in the study group (*p* < 0.001), compared to 1.9% in the control population. Similarly, the miscarriage rate was particularly high in the study group with enlarged NT and a normal karyotype compared to the control (9.2%, *p*-value < 0.001). Three cases of intrauterine foetal death were observed in the study group, representing 2.8%, while none were reported in the control group (*p*-value < 0.001). Moreover, the incidence of spontaneous preterm birth in the study group was also significantly high, 11% and 4.9% in the study and control groups, respectively (*p*-value 0.020). No cases of pregnancy termination were observed in the control population, while there were four cases in the study population, representing 3.7% with a significance of *p*-value < 0.001.

The parameters of delivery outcomes such as live birth rate, stillbirth rate, Apgar score at 1 min and presence of anomalies significantly differed between the control and study populations (Table 3). The live birth rate in the population was 89.56%, with a group breakdown that varied at 94.1% and 74.3% in the control and study groups, respectively (*p* = <0.001). There were no stillbirths in the control, while the study population had four cases (3.7%), which was significant (*p*-value < 0.001). Interestingly, there were no group differences in the neonates’ birth weight percentiles or Z-scores (*p*-values 0.444 and 0.671, respectively). The Apgar score of the neonates at 1 min was markedly lower in the study group compared to the control population (*p*-value < 0.001), and the values improved to comparable levels after 5 min of birth. The leading observation in the study was the presence of anomalies in the foetus or neonate. The observed anomaly rate in the population stood at 7.08%, significantly differing between the control and study groups. There were 21 cases of anomalies in the study group, which was substantially higher than the 12 observed in the control (*p*-value < 0.001).

### 3.3. Factors Affecting Pregnancy and Delivery Outcomes

A binary regression model for adverse pregnancy outcomes was built to understand the factors affecting adverse pregnancy outcomes in the whole population (Table 4). Based on the Cox and Snell R Square Nagelkerke R Square values, anywhere between a 10.8% to 24% change in adverse pregnancy outcome can be accounted to the predictor variables in the model such as age, BMI, parity, NT, consanguinity and the result of the second-trimester scan. The odds of having adverse pregnancy outcomes were positively correlated with age, parity, NT and consanguinity, while it was negatively associated with BMI and normal scan results (Table 5). Out of all the predictor variables entered in the model, NT and second-trimester scan results were the significant predictor variables in the model. The correlation of these factors to adverse pregnancy outcomes was also significant. Similarly, the rate of anomalies was correlated with the various delivery outcomes in the population (Table 6). There was a significant correlation between the abnormalities detected to factors such as NT, normal second-trimester scan result, birth weight, and Apgar scores of the neonates at 1 and 5 min. Factors such as normal scan results, birth weight, and Apgar scores were negatively correlated with neonate anomalies. At the same time, there was a positive linear association with NT measured during pregnancy in the neonates.

## 4. Discussion

The results of our study have provided strong evidence to support the hypothesis that enlarged NT is associated with an increased risk of adverse pregnancy outcomes, even when common aneuploidies are excluded with invasive perinatal testing. Our research has demonstrated a clear link between enlarged NT and structural or genetic anomalies and the risk of miscarriage, spontaneous preterm birth, and intrauterine foetal death. Despite these risks, our study found that a healthy neonate was still likely to be born in 87.1% of cases where NT was enlarged. This figure is comparable to the 81% reported in a previous study conducted in the Netherlands [18]. There are many recent studies showing that enlarged NT is associated with adverse pregnancy outcomes [19,20,21]. These findings highlight the importance of close surveillance of pregnancy with enlarged NT and anormal karyotype. It also underscores the need for follow up of these high-risk pregnancies in dedicated foetal maternal medicine clinics.

There are many mechanisms that have been described for the aetiology of enlarged NT in foetuses. Since many structural anomalies are associated with the heart, cardiac dysfunction is described as a cause of increased NT [22]. An investigation involving chromosomally normal foetuses with enlarged NT during the second trimester found a decrease in diastolic blood flow [23]. Importantly, it should be emphasised that even a minor disturbance in cardiac diastolic function during the first trimester can lead to an elevation in NT and abnormal ductal flow [14]. Another cause for NT enlargement is alterations to the extracellular matrix, such as the increase in hyaluronic acid in the nuchal skin of the trisomy 21 foetus [24]. Hyaluronic acid, a high-molecular-weight polysaccharide, can trap substantial quantities of solvents within the extracellular matrix [25]. In addition, the failure of lymphatic drainage is another critical cause of enlarged NT [16]. The inability of lymphatic drainage to occur can result either from irregular development of the lymphatic system, or from hindered foetal movements linked to various neuromuscular disorders [14]. Other causes include foetal anaemia [22], foetal hypoproteinaemia [26] and foetal infection [27].

Our study has provided important insights into the prevalence of anomalies in foetuses with enlarged NT and a normal karyotype. Specifically, we found that 21% of the foetuses in our study population exhibited some form of anomaly, with 85.7% of these cases involving structural abnormalities. Some of the most prominent structural anomalies observed in our population included the absence of a nasal bone, the absence of a ductus venosus, hydrops fetalis, cystic hygroma, and ambiguous genitalia and pterygium syndrome. Notably, the percentage of foetuses with structural anomalies in our study population was much higher than those reported in earlier studies. The reported structural anomaly rate was 10.6% in a similar foetal population in the Netherlands [28], 9.7% in Spain [29], 24% in Brazil [30] and 18.5% in Turkey [31].

The higher rates of anomalies in the population can be attributed to the higher rate of consanguinity. The population’s consanguinity rate in the present study stood at 35.8% (36.3% and 33.9% in the control and study groups, respectively). In a 20-year retrospective cohort study of pregnancy outcomes in a multi-ethnic population in Germany, the frequency of major anomalies among consanguineous cases was 10.9%, much higher than non-consanguineous cases (2.9%) [32]. This cohort consisted of various ethnicities, including Middle Eastern. The reported consanguinity among the Middle Eastern population was comparable to our study, 33.6%. There were 162 consanguineous cases in our study population, and anomalies were observed in 8% compared to the 6.1% reported by Becker et al. [32]. Our data are commensurable with those reported for Middle Eastern immigrants in Europe. These observations reinforce the relevance of our work due to the higher rates of consanguinity in the population. The incidents of NT enlargement and the risk of anomalies are high in the UAE population.

Another marked observation was the significantly higher rate of miscarriages and spontaneous preterm birth with enlarged NT and a normal karyotype. The foetal miscarriage rate was 9.2% in our population, significantly higher than the Netherlands cohort of 4% [18] and comparable to the 11.34% described in the Turkish study [31]. Intrauterine foetal death and stillbirth in the population were 2.8% and 3.7%, respectively, and the rate of pregnancy termination was 3.7%. These values vary considerably across different studies. For instance, in similar studies with selected or unselected populations with a control group, foetal death ranged between 0.5% and 1.9%, the miscarriage rate between 0.1% and 2.3%, and the pregnancy termination rate between 0.1% and 0.3% [15,28,33,34].

The variability in pregnancy and delivery outcomes across studies is to be expected. In most cases, these comparisons are complicated due to the lack of consensus regarding classifying anomalies. Moreover, there is no consensus on the definition of miscarriage or intrauterine/perinatal/postnatal or neonatal death. A significant strength of our study is its well-defined control population for comparing pregnancy and delivery outcomes. A major drawback of studies without a control group is that they can only report the outcome’s prevalence and do not highlight whether the observed prevalence is higher than expected from a normal population [34]. A limitation of the study was the foetuses’ lack of exome-sequencing data. Hence, a more detailed discussion of genetic disorders was not possible. The study also has a relatively small sample size, and we chose the 95th percentile as the definition of enlarged NT. Another limitation of this study is its retrospective nature, which can introduce selection bias. However, we employed three data collectors and included consecutive cases for both arms of the study to ensure randomisation. The UAE has a multi-ethnic population [35], and it would have been ideal to look at the ethnic/racial differences in enlarged NT measurements. However, this type of analysis was not possible since the health information software Astraia (NEXUS/ASTRAIA GmbH, Munich, Germany) that is used in the Maternal Foetal Medicine Clinic does not have a clear categorisation for the different ethnicities of the UAE.

NT measurement has been used as a biomarker in prenatal screening for congenital anomalies for over 40 years. Despite its long-standing use, accurately counselling parents when enlarged NT is detected with a normal karyotype remains a significant challenge [36]. Healthcare providers must carefully balance exercising caution, while avoiding undue anxiety for parents about future developments and postnatal outcomes. Anxiety about pregnancy outcomes may persist and lead to termination in some populations, even without anomalies. Therefore, data on the association between enlarged NT in cases with karyotypically normal foetuses is essential for describing the risks of adverse outcomes in pregnancy and developing effective pregnancy-management strategies. Notably, this is the first study of its kind in the UAE population, providing important insights into the challenges and opportunities for improved care in this region. With the insights from this study, it becomes feasible to assess the likelihood of intrauterine survival and the birth of a healthy baby without major defects for enlarged NT measurements in the UAE population. This information will be valuable in advising parents facing pregnancies with enlarged foetal NT and devising the right course of action for subsequent investigations.

## 5. Conclusions

In foetuses with enlarged NT and a normal karyotype, there is an increased risk of adverse pregnancy outcomes, including miscarriage, intrauterine foetal death, termination of pregnancy, spontaneous preterm birth, stillbirth and incidence of anomalies. In this study, we have provided data that can help in counselling parents about the possible risks of NT enlargement in the UAE population. Based on the observations in the study, further analysis of data from larger cohorts can give more insight into the foetal population’s characteristics for devising more suitable pregnancy management strategies.

## Figures and Tables

**Table 1:** Demographic data of control and study groups.

	Control		Study		*p*-Value
	N = 370		N = 109		
Age	32.28	±5.094	33.78	±6.139	0.0077
BMI (kg/m^2^)	28.06	±5.726	27.14	±4.594	0.2466
Parity					
<4	245	66.2%	73	67.0%	0.883
≥4	125	33.8%	36	33.0%	
Type of Conception					
Assisted	10	2.7%	1	0.9%	0.273
Spontaneous	359	97.3%	108	99.1%	
Consanguinity					0.650
No	235	63.7%	72	66.1%	
Yes	134	36.3%	37	33.9%	
Consanguinity Group					0.643
None	235	63.7%	72	66.1%	
First-degree cousin	104	28.5%	32	29.4%	
Second-degree cousin	26	7.1%	5	4.6%	
CRL (mm)	64.58	±8.719	64.63	±10.99	0.9507
NT (mm)	1.747	±0.306	4.368	±1.932	<0.0001

Categorical data are presented as N (%), and continuous data are presented as mean ± s.d. Model assumptions were checked by D’Agostino and Pearson test and Shapiro–Wilk test. Mann–Whitney test was used to compare control and study groups. All categorical data were analysed by chi-square statistics. Statistical significance set as *p*-value < 0.05. BMI—Body Mass Index, CRL—Crown–rump length, NT—Nuchal Translucency.

**Table 2:** Pregnancy outcomes in the control and study groups.

	Control		Study		*p*-Value
Second Trimester Scan Result	N = 364		N = 103		<0.001
Abnormal	7	1.9%	29	28.2%	
Normal	357	98.1%	74	71.8%	
Miscarriage (Before 22 weeks)	N = 370		N = 109		<0.001
No	366	98.9%	99	90.8%	
Yes	4	1.1%	10	9.2%	
Foetal Demise (After 22 weeks)	N = 370		N = 109		0.001
No	370	100.0%	106	97.2%	
Yes	0	0.0%	3	2.8%	
Spontaneous Preterm Birth	N = 370		N = 109		0.020
No	352	95.1%	97	89.0%	
Yes	18	4.9%	12	11.0%	
Termination of Pregnancy	N = 370		N = 109		<0.001
No	370	100%	105	96.3%	
Yes	0	0%	4	3.7%	

Data are presented as N (%). Group comparison using chi-square statistics. Statistical significance set as *p*-value < 0.05.

**Table 3:** Delivery outcomes in the control and study groups.

	Control		Study		*p*-Value
Live Birth	N = 370		N = 109		<0.001
No	22	5.9%	28	25.7%	
Yes	348	94.1%	81	74.3%	
Still Birth	N = 370		N = 109		
No	370	100%	104	96.3%	<0.001
Yes	0	0%	4	3.7%	
Birth Weight Percentile	N = 365		N = 91		0.444
≤10th	53	14.5%	16	17.6%	
11–89th	303	83.0%	71	78.0%	
≥90th	9	2.5%	4	4.4%	
Z Score	N = 365		N = 91		0.671
Severely growth restricted	82	22.5%	23	25.3%	
Moderately growth restricted	163	44.7%	36	39.6%	
Normal	120	32.9%	32	35.2%	
Apgar (1 min)	N = 365		N = 91		<0.001
<7	5	1.4%	8	8.8%	
≥7	360	98.6%	83	91.2%	
Apgar (5 min)	N = 366		N = 91		0.064
<7	3	0.8%	3	3.3%	
≥7	363	99.2%	88	96.7%	
Congenital Structural Anomalies	N = 366		N = 100		<0.001
No	354	96.7%	79	79.0%	
Yes	12	3.3%	21	21.0%	

Data are presented as N (%). Group comparison using chi-square statistics. Statistical significance set as *p*-value < 0.05.

**Table 4:** Factors affecting adverse pregnancy outcomes in the whole population.

		95% CI.		
	Odds Ratio	Lower	Upper	*p*-Value
Age	1.034	0.959	1.115	0.387
BMI	0.960	0.894	1.031	0.265
Parity	1.786	0.761	4.193	0.183
NT (mm)	1.382	1.116	1.710	0.003
Consanguinity	1.181	0.539	2.588	0.678
2nd trimesters scan (normal)	0.135	0.051	0.360	<0.001
Constant	0.162			0.253

BMI—Body Mass Index, NT—Nuchal Translucency. Results are presented as an adjusted odds ratio with 95% Confidence Interval (CI). A two-sided *p*-value of 0.05 was considered statistically significant. Potential covariates associated with adverse pregnancy outcomes adjusted for in the model included age, BMI and NT as continuous variables and consanguinity, second-trimester scan result and parity as categorical variables.

**Table 5:** Correlation for factors affecting adverse pregnancy outcomes in the population.

	Correlation Coefficient	*p*-Value
Age	0.92 *	0.045
BMI (kg/m^2^)	−0.033	0.468
Parity	0.061	0.185
NT (mm)	0.201 *	<0.001
Consanguinity	0.002	0.972
2nd-trimester scan (normal)	−0.364 *	<0.001

Statistical significance set as *p*-value < 0.05. * Significant correlation. BMI—Body Mass Index, NT—Nuchal Translucency.

**Table 6:** Spearman correlation for factors affecting the population’s delivery outcomes.

	Correlation Coefficient	*p*-Value
Age	−0.013	0.775
BMI (kg/m^2^)	0.037	0.436
Parity	−0.026	0.582
NT (mm)	0.147 *	0.002
Consanguinity	0.041	0.377
2nd-trimester scan (normal)	−0.331 *	<0.001
Birth Weight (g)	−0.156 *	<0.001
Apgar 1 min	−0.278 *	<0.001
Apgar 5 min	−0.104 *	0.026

Statistical significance set as *p*-value < 0.05. * Significant correlation. BMI—Body Mass Index, NT—Nuchal Translucency.

## Data Availability

The data supporting this study’s findings are available from the corresponding author upon reasonable request.
